# Bidirectional relationship between depression and activities of daily living and longitudinal mediation of cognitive function in patients with Parkinson’s disease

**DOI:** 10.3389/fnagi.2025.1513373

**Published:** 2025-02-12

**Authors:** Yue Xu, Durong Chen, Meiqi Dong, Yun Zhang, Hongmei Yu, Yanqing Han

**Affiliations:** ^1^Department of Neurology, Shanxi Cardiovascular Hospital, Taiyuan, China; ^2^Department of Health Statistics, School of Public Health, Shanxi Medical University, Taiyuan, China; ^3^Department of Neurology, Cardiovascular Hospital Affiliated to Shanxi Medical University, Taiyuan, China; ^4^Shanxi Provincial Key Laboratory of Major Diseases Risk Assessment, Shanxi Medical University, Taiyuan, China; ^5^MOE Key Laboratory of Coal Environmental Pathogebicity and Prevention, Shanxi Medical University, Taiyuan, China

**Keywords:** Parkinson’s disease, depression, cognitive function, activities of daily living, cross-lagged panel model, longitudinal mediation analysis

## Abstract

**Objective:**

To investigate the bidirectional relationship between depression and activities of daily living (ADL) in Parkinson’s disease (PD) patients and explore the mediating role of cognitive function over time.

**Methods:**

Data from 892 PD patients from the Parkinson’s Progression Markers Initiative (PPMI) database were included in this study, and depression, cognitive function, and ADL were measured using the Geriatric Depression Scale (GDS-15), Montreal Cognitive Assessment Scale (MoCA), and Unified Parkinson’s Disease Rating Scale, Part II (UPDRS II) respectively. The cross-lagged panel model (CLPM) was employed to analyze the reciprocal relationship between depression and ADL. Then, we explored the mediating role of cognitive function in the bidirectional relationship between depression and ADL in patients with PD, and the mediation effect test was carried out using a bias-corrected nonparametric percentile bootstrap approach.

**Results:**

Depression in patients with PD predicted their subsequent ADL (*β* = 0.079, *p* < 0.01), and ADL also predicted their subsequent depression (*β* = 0.069, *p* < 0.05), In addition, Bootstrap analysis showed that cognitive function played a significant mediating role in prediction of depression to ADL in patients with PD (*β* = 0.006, *p* = 0.074, 95%CI = 0.001 ~ 0.014), and cognitive function also played a significant mediating role in prediction of depression to ADL (*β* = 0.006, *p* = 0.067, 95%CI = 0.001 ~ 0.013).

**Conclusion:**

There is a bidirectional relationship between depression and ADL in patients with PD. Furthermore, we found that cognitive function mediates the relationship that exists between depression and ADL in patients with PD. Interventions aimed at enhancing cognitive function could potentially lessen the vicious cycle of depression and ADL in PD, thus improving patient quality of life (QOL).

## 1 Introduction

Parkinson’s disease (PD) is the second most prevalent neurological disorder after Alzheimer’s. As global aging advances, its incidence is increasing, particularly affecting older individuals, which places a greater burden on societal and economic structures (Dorsey et al., 2018; Santos García et al., 2019). While PD patients commonly exhibit motor symptoms, non-motor symptoms such as depression, cognitive impairment, and autonomic dysfunction often manifest earlier. These symptoms can significantly impact the quality of life (QOL) more profoundly than motor symptoms (Hussein et al., 2021).

One of the most prevalent non-motor symptoms among people with PD is depression, which usually manifests in the beginning phases of the illness, and affects approximately 40–50% of patients (Camargo et al., 2017; Cong et al., 2022). Activities of Daily Living (ADL) are crucial indicators of PD severity and directly reflect patients the QOL (Santos García et al., 2019). Several research have examined a link between depression and ADL in PD patients. Several cross-sectional studies have discovered that depression is a strong predictor of ADL, and depression is inversely linked with ADL in patients with PD (Lawrence et al., 2014; He et al., 2021a). Additionally, longitudinal research has shown that lower ADL scores are a risk factor for depression in PD patients (Antar et al., 2021). However, the studies mentioned above all explored the unidirectional relationship between depression and ADL in PD patients. Although a recent cohort study showed a bivariate relationship between depression and both ADL and instrumental activities of daily living (IADL) in older adults, this study included ADL in addition to IADL, and different scales were used to assess ADL (Zhu et al., 2024).

Another prevalent non-motor symptom of PD is cognitive impairment, about 25% of people with PD are diagnosed with mild cognitive impairment at initial diagnosis, and up to 80% experience Parkinson’s disease dementia (PDD) within 15–20 years of diagnosis (Hely et al., 2008; Litvan et al., 2011). It has been shown that depression in PD patients is a risk factor for cognitive impairment, and that cognitive impairment in PD patients is associated with ADL difficulties (Rosenthal et al., 2010; Hemphill et al., 2023), In a cross-sectional study, Jones et al. assessed 214 US patients and found that those with Parkinson’s Disease Mild Cognitive Impairment (PD-MCI) exhibited more severe depression than those without cognitive deficits (Jones et al., 2016). And impaired ADL in PD patients increase the risk of cognitive impairment (Jones et al., 2016; D’Iorio et al., 2018).

The above studies are two-by-two relationship between depression, ADL, and cognitive function in PD patients. There are some studies on the relationship between cognitive function, ADL, and depression. Sun et al. found that cognitive function can influence depressive status through ADL in older adults (Sun et al., 2024). Ai et al. found a significant interaction between ADL limitation and cognitive dysfunction in older adults, both of which are risk factors for depression (Ai et al., 2024). However, these studies were conducted on older adults, not PD patients. One Korean study of 32 MCI patients found that cognitive function mediated the effect of depression on ADL (Jung et al., 2023). However, this study was a cross-sectional study and could not explore the causal relationship between variables. Survey-based research often employ Cross-lagged panel model (CLPM) to establish causality because it can eliminate autoregressive effects (Schuurman et al., 2016). The examination of the interaction between variables has been applied widely (Cole and Maxwell, 2003).

Our study analyzed the relationship between cognitive function, ADL, and depression in PD patients to fill the gap in longitudinal research on the relationship between depression, cognitive function, and ADL in PD patients. This study explored the bidirectional relationship between depression and ADL. Then, we also investigated the longitudinal mediating role of cognitive function in the bidirectional relationship between depression and ADL in PD patients. Patients with PD presenting with depression can have an impact on their QOL by affecting their motor symptoms (Nagarajan et al., 2024). PD patients with depression affect their ADL, which serious affects QOL (Cong et al., 2022). Depression and ADL in PD patients can mediate the impact of their cognitive function on the QOL (He et al., 2021a). So as to provide theoretical basis for enhancing the QOL of PD patients.

## 2 Methods

### 2.1 Data and sample

**Figure 1:**
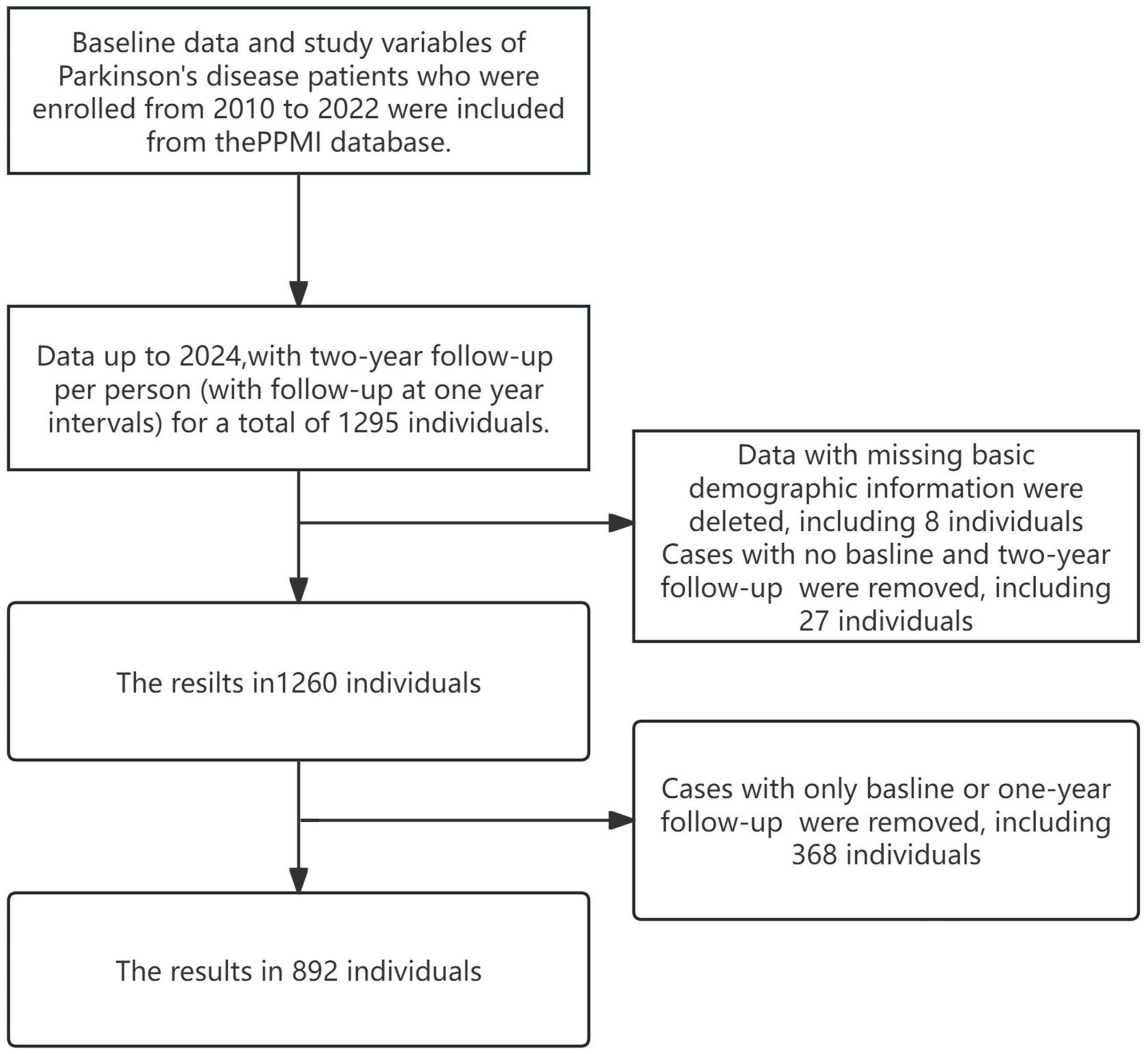
Sample selection.

Data used in the preparation of this article were obtained (on January 29, 2024) from the Parkinson’s Progression Markers Initiative (PPMI) database,^1^ RRID:SCR_006431. For up-to-date information on the study, visit http://www.ppmi-info.org. PPMI is an extensive global, multicenter, longitudinal observational study. In order to better understand the etiology and progression of the disease, a longitudinal clinical, imaging, and biomarker assessment was carried out at 21 clinical sites in the US, Europe, and Australia using standardized data collecting. We chose data from the PPMI database from 2010 to 2024 at baseline and at two-year follow-up (with follow-up at 1-year intervals), which includes PD diagnoses. Participants with only baseline data or one-year follow-up, missing data from both the baseline and two-year follow-up, or lacking essential demographic information were excluded. Figure 1 shows the specific sample inclusion criteria, and our ultimate sample size was 892 people. None of our participants received any therapy at baseline. However, they underwent confirmatory tests, including clinical and cognitive assessments, imaging examinations, and biological specimen collection, all approved by the local Central Institutional Review Board. Each participant provided written informed consent prior to enrollment.

### 2.2 Measurements

Depression was assessed using the Geriatric Depression Scale (GDS). We selected the GDS-15, a shorter version with demonstrated reliability and validity in clinical assessments (Acosta Quiroz et al., 2020). The total score ranged from 0 to 15, with ≥5 indicating mild depression and ≥10 indicating moderate to severe depression.

ADL were assessed using the UPDRS II, the second part of the Unified Parkinson Disease Rating Scale, consisting of 13 items and a score range of 0–52. Higher scores indicate poorer functionality (Goetz et al., 2007). The UPDRS II has been shown to have good reliability and validity in patients with PD (Ramsay et al., 2020), and this is a useful marker for tracking the progression of the illness.

The Montreal Cognitive Assessment (MoCA) was used to assess cognitive function, which consists of 11 items with scores ranging from 0 to 30 and ≥26 normal. It has high sensitivity and specificity in the detection of cognitive function (Nasreddine et al., 2005).

### 2.3 Covariates

Including the age (<56 years, 56–65 years, and >65 years), gender (female, male), education (<13 years, 13–23 years, and >23 years), race white, black, Asian, and other, family history (1st degree family, no 1st degree family, and no family), and duration of disease (<5 years, 5–10 years, and >10 years) in patients with PD. In addition, patients with PD recorded in their medical history the use of levodopa in dopaminergic drug therapy during the follow-up period, indicated as Levodopa Equivalent Daily Dose (LEDD). LEDD1 indicates first year follow-up Levodopa Equivalent Daily Dose and LEDD2 indicates second year follow-up Levodopa Equivalent Daily Dose.

### 2.4 Statistical analysis

Data analysis was conducted using SPSS27.0 Missing data were imputed using multiple interpolation techniques. Quantitative data were expressed as mean ± standard deviation, and qualitative data were reported as frequencies (percentage). Independent samples t-test and one-way ANOVA were used to compare the scores of depression, cognitive function, and ADL across patients with different characteristics. Pearson correlation analysis was utilized to estimate relationships between variables. Mplus8.3 was used to fit the CLPM for depression, cognitive function, and ADL. *α* = 0.05 was used as the test level. Holographic great likelihood estimation was used for parameter estimation, and the mediation effect was tested using the bias-corrected nonparametric percentile Bootstrap 1,000 iterations were taken (Holm et al., 2021; Yang and Zhao, 2024). And the coefficient product was significant and the mediation effect was significant if the confidence interval did not contain zero. The fit of the model was evaluated using several indices: chi-square/degrees of freedom (*χ*^2^/df), Comparative Fit Index (CFI), Tucker-Lewis Index (TLI), Root Mean Square Error of Approximation (RMSEA), and Standardized Root Mean Square Residual (SRMR). A good model fit was indicated by (*χ*^2^/df) < 5, CFI and TLI > 0.90, and RMSEA and SRMR < 0.08 (Bentler, 1990; Hu and Bentler, 1999; Schermelleh-Engel et al., 2003).

## 3 Results

### 3.1 Basic information on the subject of the study

This study included 892 PD patients in total, 551 (61.8%) of them were male and 341 (38.2%) were female, most of them were white (93.6%), and most of them had 13–23 years of education (80.3%). Age > 65 years was 45.3%, and disease duration of <5 years was 92.6%, The percentage of LEDD1 > 500 mg in the first year of follow-up was 77.6%, and the percentage of LEDD2 > 500 mg in the second year of follow-up was 63.1%, as shown in Table 1.

**Table 1:** Demographic information on the study population (*N* = 892).

Variables	*N* (%)
Gender	
Female	341 (38.2%)
Male	551 (61.8%)
Education (years)	
<13	160 (17.9%)
13–23	716 (80.3%)
>23	16 (1.8%)
Race	
White	835 (93.6%)
Black	14 (1.6%)
Asian	14 (1.6%)
Other	29 (3.3%)
Family history	
1st degree family	205 (23.0%)
No-1st degree family	111 (12.4%)
No family	576 (64.6%)
Age (years)	
<56	229 (25.7%)
56–65	259 (29.0%)
>65	404 (45.3%)
Duration of disease (years)	
<5	826 (92.6%)
5–10	64 (7.2%)
>10	2 (0.2%)
LEDD1 (mg)	
<500	692 (77.6%)
500–1,000	156 (17.5%)
>1,000	44 (4.9%)
LEDD2 (mg)	
<500	563 (63.1%)
500–1,000	249 (27.9%)
>1,000	80 (9.0%)

Between-group comparisons of baseline ADL scores among demographic subgroups revealed statistically significant differences within age and disease duration groups (*p* < 0.05). Between-group comparisons of patients’ depression scores at baseline based on demographic subgroups showed statistically significant differences in baseline patients’ depression scores within education level, race, and disease duration groups (*p* < 0.05). Comparisons of baseline cognitive function scores among demographic subgroups demonstrated statistically significant differences within educational level, racial, family history, age, and disease duration groups (*p* < 0.05), as shown in Table 2.

**Table 2:** Study participants’ scores on Baseline ADL, depression, and cognitive function.

Variables	Mean ± SD			*t/F*			*p*		
	UPDRSII (Max score: 30)	GDS (Max score: 14)	MOCA (Max score: 30)	UPDRSII	GDS	MOCA	UPDRSII	GDS	MOCA
Gender									
Female	6.26 ± 4.56	2.71 ± 2.88	26.79 ± 2.89	−0.384	1.598	1.607	0.701	0.11	0.108
Male	6.38 ± 4.67	2.41 ± 2.61	26.48 ± 2.75						
Education (years)									
<13	6.16 ± 4.51	2.94 ± 2.86	25.80 ± 3.52	0.517	3.275	8.115	0.596	0.038	<0.001
13–23	6.34 ± 4.63	2.46 ± 2.70	26.76 ± 2.61						
>23	7.38 ± 5.68	1.50 ± 1.67	27.19 ± 1.94						
Race									
White	6.33 ± 4.66	2.45 ± 2.67	26.69 ± 2.75	0.049	5.273	5.923	0.986	0.001	<0.001
Black	6.71 ± 4.76	4.71 ± 4.21	25.71 ± 3.15						
Asian	6.29 ± 3.93	2.79 ± 2.42	26.14 ± 3.11						
Other	6.14 ± 4.07	3.72 ± 2.80	24.59 ± 3.45						
Family history									
1st degree family	6.76 ± 4.98	2.75 ± 2.74	25.74 ± 3.43	1.396	0.927	12.978	0.248	0.396	<0.001
No-1st degree family	5.91 ± 4.07	2.41 ± 2.56	27.05 ± 2.32						
No family	6.26 ± 4.59	2.47 ± 2.72	26.81 ± 2.58						
Age (years)									
<56	6.10 ± 4.78	2.80 ± 2.95	27.31 ± 2.80	4.482	2.717	25.131	0.012	0.067	<0.001
56–65	5.77 ± 4.24	2.24 ± 2.75	27.06 ± 2.22						
>65	6.82 ± 4.72	2.56 ± 2.54	25.89 ± 2.99						
Duration of disease (years)									
<5	6.14 ± 4.40	2.43 ± 2.64	26.70 ± 2.65	12.137	9.896	8.545	<0.001	<0.001	<0.001
5–10	9.21 ± 6.62	4.05 ± 3.41	25.16 ± 4,23						
>10	4.00 ± 2.83	1.00 ± 1.41	26.59 ± 2.81						

GDS, Geriatric Depression Scale score; MOCA, Montreal Cognitive Assessment Scale score; UPDRS II, Unified Parkinson’s Disease Rating Scale Part II score.

Between-group comparisons of patients’ ADL scores, depression scores, and cognitive function scores at the first year of follow-up were made according to LEDD subgroups (*p* < 0.001), as shown in Table 3.

**Table 3:** Study participants’ scores on ADL, depression, and cognitive function in the first year follow-up.

Variables	Mean + SD			*F*			*p*		
	UPPRSII	GDS	MOCA	UPDRSII	GDS	MOCA	UPDRSII	GDS	MOCA
LEDD1									
<500 mg	7.16 ± 4.82	2.38 ± 2.74	26.51 ± 2.88	15.210	17.386	9.233	<0.001	<0.001	<0.001
500–1,000 mg	9.28 ± 7.03	3.73 ± 3.58	25.32 ± 4.43						
>1,000 mg	10.20 ± 6.37	3.95 ± 3.72	25.64 ± 4.44						

GDS, Geriatric Depression Scale score; MOCA, Montreal Cognitive Assessment Scale score; UPDRS II, Unified Parkinson’s Disease Rating Scale Part II score; LEDD1, first year follow-up Levodopa Equivalent Daily Dose.

Comparison of the patients’ ADL scores, depression scores and cognitive function scores at the second year follow-up was made between groups according to LEDD subgroups, and the results showed that the differences of ADL scores and depression scores at the second year follow-up were statistically significant within LEDD subgroups (*p* < 0.001), and the differences of the patients’ cognitive function score at the second year follow-up were statistically significant within LEDD subgroups (*p* < 0.05), as shown in Table 4.

**Table 4:** Study participants’ scores on ADL, depression, and cognitive function in the second year follow-up.

Variables	Mean + SD			*F*			*p*		
	UPDRSII	GDS	MOCA	UPDRSII	GDS	MOCA	UPDRSII	GDS	MOCA
LEDD2									
<500 mg	7.45 ± 5.43	2.49 ± 2.70	26.53 ± 3.25	13.972	8.823	4.449	<0.001	<0.001	0.012
500–1,000 mg	8.73 ± 6.83	3.00 ± 3.05	26.37 ± 3.52						
>1,000 mg	11.03 ± 7.38	3.83 ± 3.59	25.30 ± 4.40						

GDS, Geriatric Depression Scale score; MOCA, Montreal Cognitive Assessment Scale score; UPDRS II, Unified Parkinson’s Disease Rating Scale Part II score; LEDD2, second year follow-up Levodopa Equivalent Daily Dose.

### 3.2 Correlates of depression, ADL and cognitive function

Table 5 presents the mean and standard deviation of the scores for depression, cognitive function, and ADL at baseline and at two-year follow-up assessments. It also showed the interrelationships among these variables via correlation coefficients. The Pearson correlation analysis indicated that depression was positively correlated with ADL (*p* < 0.01), while both depression and ADL were negatively associated with cognitive function (*p* < 0.01).

**Table 5:** Correlation analysis among variables.

Variables	1	2	3	4	5	6	7	8	9
GDS1	1								
GDS2	0.642^**^	1							
GDS3	0.609^**^	0.704^**^	1						
MOCA1	−0.124^**^	−0.141^**^	−0.185^**^	1					
MOCA2	−0.188^**^	−0.205^**^	−0.251^**^	0.627^**^	1				
MOCA3	−0.180^**^	−0.234^**^	−0.283^**^	0.609^**^	0.790^**^	1			
UPDRSII1	0.328^**^	0.297^**^	0.298^**^	−0.142^**^	−0.194^**^	−0.222^**^	1		
UPDRSII2	0.299^**^	0.365^**^	0.349^**^	−0.183^**^	−0.254^**^	−0.310^**^	0.682^**^	1	
UPDRSII3	0.258^**^	0.318^**^	0.367^**^	−0.198^**^	−0.283^**^	−0.338^**^	0.637^**^	0.772^**^	1
Mean	2.53	2.70	2.75	26.59	26.26	26.37	6.33	7.68	8.13
SD	2.72	3.01	2.91	2.80	3.32	3.46	4.62	5.44	6.12

GDS, the Geriatric Depression Scale score; MOCA, Montreal Cognitive Assessment Scale score; UPDRS II, Unified Parkinson’s Disease Rating Scale Part II score.

** *p < 0.01.*

### 3.3 Longitudinal mediation analysis of cognitive function in the relationship between depression and ADL in patients with PD

**Figure 2:**
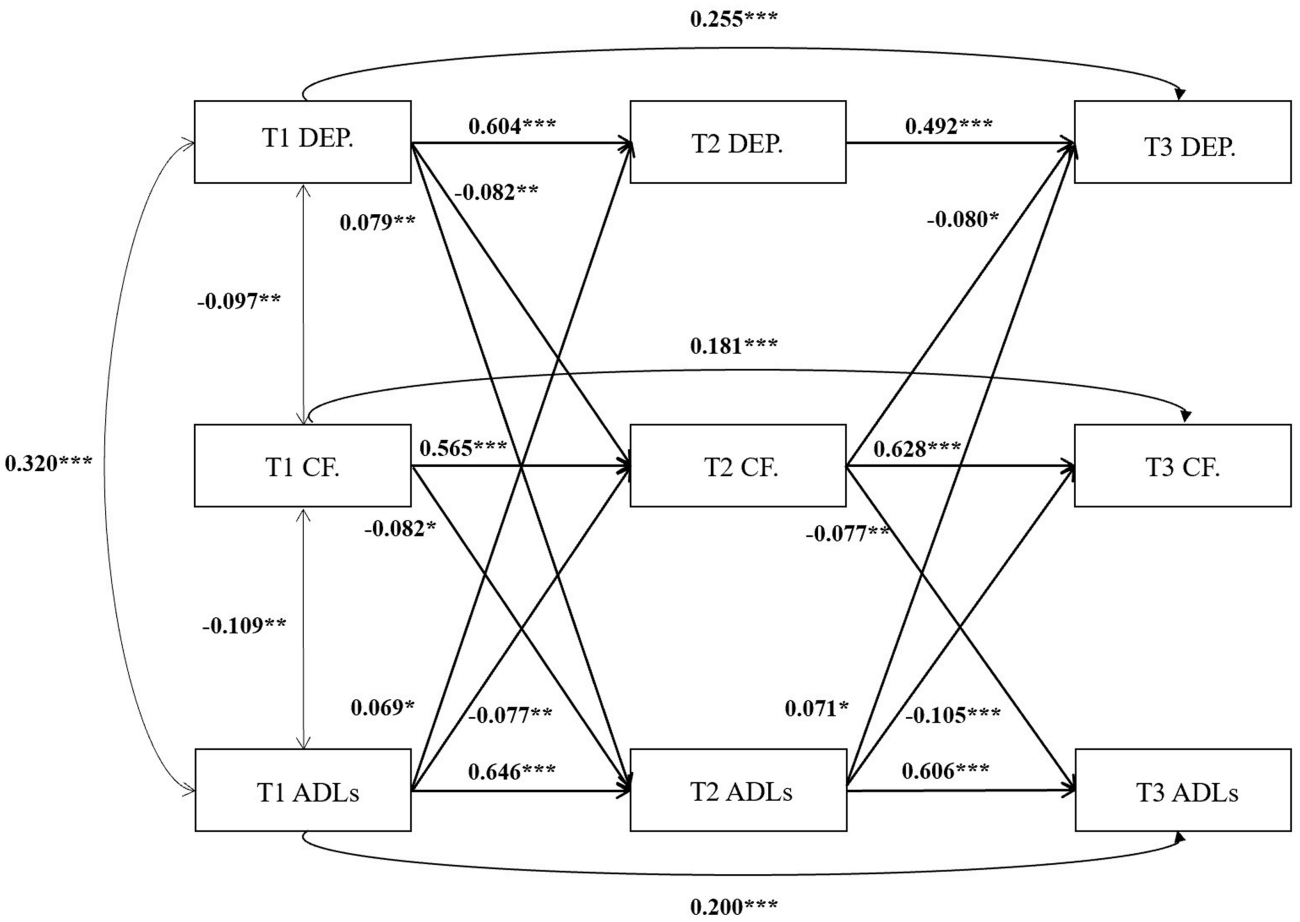
Cross-lagged panel model of depression, cognitive function, and ADL. *p < 0.05, **p < 0.01, ***p < 0.001, ADL, Activities of daily living; CF, Cognitive Function; DEP: Depression; T1, Baseline; T2, First year follow-up; T3, Second year follow-up.

To evaluate the longitudinal mediating role of cognitive function in the relationship between depression and ADL, we constructed the CLPM incorporating three time points. This model adjusted for factors including age, gender, education, race, family history, and disease duration and LEDD in PD patients, positioning depression as the independent variable, cognitive function as the mediator, and ADL as the dependent variable. After stepwise removal of non-significant paths, the final results are shown in Figure 2. Model fit indices were favorable, with *χ*^2^/df = 3.10, CFI = 0.979, TLI = 0.946, RMSEA = 0.049, and SRMR = 0.045. T1 depression significantly predicted T2 ADL (*β* = 0.079, *p* < 0.01), T1 ADL significantly predicted T2 depression (*β* = 0.069, *p* < 0.05), which can indicate that, depression and ADL are causative of each other, and they can predict each other. T1 depression significantly predicted T2 cognitive function (*β* = −0.082, *p* < 0.01), T2 cognitive function significantly predicted T3 ADL (*β* = −0.077, *p* < 0.01). The results of Bootstrap analysis showed a significant mediating effect of T2 cognitive function (*β* = 0.006, *p* = 0.074, 95%CI = 0.001 ~ 0.014) in prediction of T1 depression on T3 ADL. T1 ADL significantly predicted T2 cognitive function (*β* = −0.077, *p* < 0.01), T2 cognitive function significantly predicted T3 depression (*β* = −0.080, *p* < 0.05). The results of Bootstrap analyses showed a significant mediating effect of T2 cognitive function (*β* = 0.006, *p* = 0.067, 95%CI = 0.001 ~ 0.013) in prediction of T1 ADL on T3 depression.

## 4 Discussion

This study utilized a longitudinal research design and focused on the relationship between depression, cognitive function, and ADL in patients with PD, with special attention to the mediating role of cognitive function. We confirmed a bidirectional relationship between depression and ADL in PD patients. Mediation analysis showed that cognitive function mediated the bidirectional relationship between depression and ADL in PD patients.

The present study provided evidence for a bidirectional relationship between depression and ADL in PD patients, which complements studies showing that PD patients’ ADL predicted subsequent depression (Xu et al., 2015; Antar et al., 2021) and that PD patients’ depression predicted subsequent ADL (Randver, 2018; Jin et al., 2020). Elderly people with limited ADL are in great need of help and care from others, their QOL is lower than before, they have fewer opportunities for social support, and they have difficulty in regulating their negative emotions in a timely manner. In turn depression can lead to fatigue, decreased energy levels and disrupted sleep patterns, further hindering the ability to perform basic daily tasks and affecting ADL (Pearson et al., 2023). It is that although the results of a recent longitudinal study showed that ADL in PD patients can influence the progression of depression, the reverse pathway could not confirm that depression in PD patients influences ADL (Wang et al., 2024), which is contradictory to the results of our findings, and the reason for this analysis may be due to the relatively large sample size of our study as well as the fact that we adjusted for a number of factors such as age and LEDD. In comparison with the study by Sperens et al. (2020), our study only found that baseline depression predicted ADL, which may have been related to the fact that the patients received treatment during the follow-up period and the shorter follow-up period of the current study.

Our study found that depression went on to affect ADL by affecting cognitive function. Depression predicted subsequent cognitive function in patients may be related to Locus coeruleus (LC). LC is considered to be the main noradrenergic nucleus in the CNS, and the accumulation of synaptic nuclear proteins in the LC leads to the loss and degeneration of LC neurons, which may result in reduced innervation of LC target nuclei and reduced levels of NE in several regions of the brain in patients with PD, leading to the onset of depression, which further reduces responsiveness to sensory inputs and impairs cognitive flexibility and vigilant attention (Zarow et al., 2003; Paredes-Rodriguez et al., 2020). Cognitive function in PD patients predicted subsequent ADL. The likely reason for this is that cognitive decline not only impairs older adults in areas such as memory and processing speed, but also reduces their sense of self-efficacy, which ultimately affects ADL (Sun et al., 2024). Therefore, depressive symptoms in older adults reduce mental flexibility and their ability to consolidate and retrieve memories, thus affecting overall cognitive function (Yatawara et al., 2016), which in turn can lead to reduced memory, difficulty in decision making, and impaired executive function can affect the patient’s independence and ability to perform daily tasks, thus affecting ADL. Additionally, depression in PD patients can affect cognitive function by affecting their non-motor symptoms of apathy, and when their cognitive function declines, it can also affect ADL by affecting apathy (Szymkowicz et al., 2022; Cui et al., 2024). Therefore, apathy also plays a role in the relationship between depression, ADL, and cognitive function, and could be included in future studies to examine apathy.

Furthermore, our study found that ADL went on to affect depression by influencing cognitive function in patients with PD. ADL predicted subsequent cognitive function, the reason for this may be that people with PD have limited ADL, and this physical helplessness increases the risk of cognitive deficits. Cognitive function in PD patients predicted subsequent depression. Interpretation of PD in terms of its neuropathologic features, especially the deterioration of the striatum, loss of dopamine and disruption of the cortico-striatal pathway, may first affect cognitive function and then increase anxiety and depression (Weintraub et al., 2005), these brain regions are affected in PD and play an important role in cognitive function as well as in emotion regulation and motivation (Lago et al., 2017). Cognitive function can mediate the interpretation of the effects of ADL on depression is complex. Elderly people’s body functions are affected by ageing, which inevitably leads to a decline in ADL levels, further leading to further cognitive impairment, and they have difficulty in dealing with a variety of situations in daily life, such as memorizing, learning, and problem solving, which can increase frustration and helplessness, thus aggravating depression (Hammar et al., 2022). Additionally, difficulties with ADL in PD patients lead to higher levels of stigma, a decrease in their social engagement interactions, which are an important way of obtaining information and social support, which in turn leads to cognitive dysfunction with lower executive function, exacerbating the development of depressive (Eccles et al., 2023; Zhang and Yang, 2024).

Since there is a bidirectional relationship between depression and ADL in PD patients, both the individual’s ADL and depression may be addressed in PD patients. Clinicians can follow up with PD patients on a regular basis, distribute depression scales for testing, and conduct psychological counseling, relaxation training, and sleep interventions for patients with depressive symptoms, thus further reducing the burden of disabling illnesses brought about by depression. When a decline in ADL has occurred, PD patients should be helped to cope positively to avoid exacerbating depressive symptoms. Given that cognitive function mediates the bidirectional relationship between depression and ADL in PD patients, special attention should be paid to improving cognitive function in PD patients. In addition to administering conventional pharmacological treatments, mainly cholinesterase inhibitors such as carbapenems and doxorubicin that have been shown to be useful in clinical practice (Rolinski et al., 2012; Seppi et al., 2019), clinicians can also inform patients that they can engage in appropriate physical exercise and cognitive training, which may have a beneficial effect on cognitive function in patients with PD (Weintraub et al., 2022).

PD patients in this study were predominantly white, older, and more educated, which is related to the criteria PPMI chose for inclusion of PD patients, and there is now recent research that suggests that ethnic minority groups such as Latinos or Hispanics have more severe non-motor symptoms such as depression and cognitive deficits compared to whites, which may have a greater impact on ADL (Duarte Folle et al., 2023), which may be due to their low economic income, are less likely to visit an outpatient neurologist, and do not receive timely treatment, leading to the increasing severity of these non-motor symptoms (Saadi et al., 2017). Therefore, future studies could examine the relationship between depression, ADL and cognitive function in PD patients of different races.

The findings of this study offer theoretical and practical insights into mitigating the negative cycle of depression and ADL in PD patients. In theory, the model explains the correlation mechanism between depression, ADL, and cognitive function, which lays the foundation for the next step of the study. In practice, the results have guiding significance for improving the vicious cycle of depression and ADL in PD patients, and intervention on cognitive function might be an appropriate choice to alleviate the vicious cycle of depression and ADL in PD patients.

Despite its strengths, the study has several limitations. Primarily, the study population was predominantly white, with most participants hailing from North America and Europe, which may limit the generalizability of the findings, given the global diversity represented in the PPMI database (Duarte Folle et al., 2023). In the future, we can study the relationship between depression, cognitive function, and ADL in PD patients of other races. Although our study adjusted for factors such as age, LEDD, etc. However, the use of antidepressants in patients with PD was not taken into account, as well as the possible influence of other non-motor symptoms such as apathy, which could be further included for analysis in the future. Finally, our study was selected as a longitudinal study with data from baseline and two-year follow-up, and although three time points were required to set up to satisfy the CLPM, more in-depth studies could be considered in the future by increasing the number of follow-up and the duration of the study (Cole and Maxwell, 2003; Little, 2024).

## 5 Conclusion

Individuals with PD exhibit a reciprocal relationship between depression and ADL, with cognitive function serving as a mediator. Given the interdependence of these variables, interventions aimed at improving cognitive function could potentially lessen the vicious cycle of depression and ADL in PD, thus improving patient the QOL. Additionally, it is essential to explore other potential pathways between ADL and depression to significantly improve the QOL in PD patients.

## Data Availability

The raw data supporting the conclusions of this article will be made available by the authors, without undue reservation.

## Footnotes

^1^ https://www.ppmi-info.org/access-data-specimens/download-data
